# Did the socioeconomic inequalities in avoidable and unavoidable mortality worsen during the first year of the COVID-19 pandemic in Korea?

**DOI:** 10.4178/epih.e2023072

**Published:** 2023-08-03

**Authors:** Rora Oh, Myoung-Hee Kim, Juyeon Lee, Rangkyoung Ha, Jungwook Kim

**Affiliations:** 1 Department of Public Health, Graduate School of Public Health, Seoul National University, Seoul, Korea; 2 Center for Public Health Data Analytics, National Medical Center, Seoul, Korea; 3 Social and Behavioural Health Sciences Division, Dalla Lana School of Public Health, University of Toronto, Toronto, ON, Canada; 4 Department of Social Welfare, Seoul National University, Seoul, Korea

**Keywords:** COVID-19, Mortality, Health inequalities, Korea

## Abstract

**OBJECTIVES:**

This study examined changes in socioeconomic inequalities in mortality in Korea before and after the outbreak of coronavirus disease 2019 (COVID-19).

**METHODS:**

From 2017 to 2020, age-standardized mortality rates were calculated for all-cause deaths, avoidable deaths (preventable deaths, treatable deaths), and unavoidable deaths using National Health Insurance claims data and Statistics Korea’s cause of death data. In addition, the slope index of inequality (SII) and the relative index of inequality (RII) by six income levels (Medical Aid beneficiary group and quintile of health insurance premiums) were computed to analyze the magnitude and change of mortality inequalities.

**RESULTS:**

All-cause and avoidable mortality rates decreased steadily between 2017 and 2020, whereas unavoidable mortality remained relatively stable. In the case of mortality inequalities, the disparity in all-cause mortality between income classes was exacerbated in 2020 compared to 2019, with the SII increasing from 185.44 to 189.22 and the RII increasing from 3.99 to 4.29. In particular, the preventable and unavoidable mortality rates showed an apparent increase in inequality, as both the SII (preventable: 91.31 to 92.01, unavoidable: 69.99 to 75.38) and RII (preventable: 3.42 to 3.66, unavoidable: 5.02 to 5.89) increased.

**CONCLUSIONS:**

In the first year of the COVID-19 pandemic, mortality inequality continued to increase, although there was no sign of exacerbation. It is necessary to continuously evaluate mortality inequalities, particularly for preventable and unavoidable deaths.

## GRAPHICAL ABSTRACT

[Fig f3-epih-45-e2023072]

## INTRODUCTION

In general, Korea’s response to the coronavirus disease 2019 (COVID-19) pandemic has been extensively praised [1-4]. Among various indicators, excess mortality is regarded as an objective estimate of the pandemic’s total impact since it includes both direct and indirect deaths caused by COVID-19 [5]. The number of excess deaths from the COVID-19 pandemic in 2020 was estimated at around 4.48 million people worldwide (95% confidence interval [CI], 3.93 to 5.07) [6]. However, during the first year of the pandemic, significant excess mortality was not observed in Korea [6-10].

Although the first year of the pandemic did not affect overall excess mortality, the impacts of the pandemic may have varied significantly across socioeconomic groups. In the United States, ethnic minority groups showed higher levels of excess mortality [11-13], while in the United Kingdom, the poorest quintile regions showed the highest excess mortality [14] and the most years of life lost [15]. In contrast, in Belgium, excess mortality was salient among high-income men of working age, first-generation non-Belgian migrant men, and low-educated women, indicating that socioeconomic inequality is not straightforward; in particular, leisure or work-related travel, which increases the risk of COVID-19 infection, might have been more common among high-income men aged 25-64 [16].

In Korea, Kim et al. [9] found excess deaths among male Medical Aid beneficiaries (531 per 100,000 population). Meanwhile, Oh et al. [10] found a disproportionate impact of the pandemic on mortality by cause of death, education level, and marital status. The excess mortality related to the pandemic was pronounced in people with metabolic and ill-defined diseases, those with lower levels of education, and those who were single [10]. Kim et al. [17] divided the Korean population into 3 income groups according to the level of national health insurance premiums and examined the relationship of income with COVID-19 infection, morbidity, and mortality. The results showed that people with low-income levels were more vulnerable to COVID-19 infection, but COVID-19 morbidity and mortality in the Korean population were unrelated to income level [17]. Thus, the consequences of socioeconomic inequality in death during the pandemic in Korean society also seem mixed.

We sought to supplement and expand upon the existing studies in 2 aspects. First, several studies have examined which causes of death have significantly increased in Korea during the COVID-19 pandemic [9,10,17], but the concept of avoidable death has not been utilized. Avoidable death refers to deaths resulting from causes that can be avoided by adequate and timely prevention and treatment [18]. Thus, this parameter can be used to indicate whether the Korean healthcare system and socioeconomic policies during the COVID-19 pandemic have adequately protected people from dying from avoidable causes. Avoidable deaths are divided into treatable and preventable ones; treatable mortality includes death that can be avoided if quality healthcare services, including timely treatment, are provided based on current medical knowledge and technology, while preventable mortality indicates deaths that can be avoided through effective public policies and primary preventive interventions [19]. Unavoidable or non-avoidable mortality refers to all causes of death except avoidable causes. By classifying all-cause deaths into avoidable (preventable and treatable) and non-avoidable deaths, we can view changes in mortality rates from various angles, with implications for the healthcare system and policy response.

Second, there seems to be little evidence of whether the pattern and magnitude of socioeconomic inequality changed during the first year of the COVID-19 pandemic. Since the focus of previous studies has been on changes in the magnitude of mortality, it is necessary to study changes in the degree of inequality as well. Our study aimed to investigate the impact of the COVID-19 pandemic on socioeconomic inequality in mortality by examining changes in absolute and relative inequality of all-cause, avoidable (preventable and treatable), and unavoidable death over the 3 years preceding COVID-19 and the first year of the pandemic.

## MATERIALS AND METHODS

### Data

We obtained cause of death data for the years 2017 to 2020 from Statistics Korea and linked it to National Health Insurance claims data, covering the entire population of Korea. The total number of deaths in the merged dataset was 1,189,022. The number of deaths included in the final analysis was 1,170,909 (Supplementary Material 1), excluding cases with missing key information such as cause of death, gender, age, and health insurance contribution.

### Mortality and causes of deaths

We calculated annual age-standardized mortality rates (ASMRs) per 100,000 using the direct standardization method with the standard 2005 mid-year population. Avoidable mortality, which is divided into treatable and preventable mortality, was defined based on the Organization for Economic Cooperation and Development (OECD)/Eurostat standardization. Some causes of death belong to both treatable and preventable mortality, so the sum of treatable and preventable mortality is greater than the total value of avoidable mortality. Meanwhile, the upper age limit was set at 74 years [19].

The list of avoidable causes of death should be updated in a timely manner to reflect advances in medical knowledge and skills and changes in the social environment [18]. Causes of death previously considered unavoidable can now be defined as avoidable. In this study, the latest version (published in 2022) of the list jointly adopted by the OECD and Eurostat was used [19], with slight modifications. For privacy protection, Statistics Korea provided some causes of death (as International Classification of Disease, 10th revision [ICD-10] codes) in aggregate form, so we could not accurately apply the OECD/Eurostat classification. The final version was developed with reference to the classifications of the United Kingdom’s Office for National Statistics [20] and Kang et al. [21], and in consultation with experts. The final classification scheme can be found in Supplementary Material 2.

### Inequality measures

Socioeconomic position (SEP) was measured by health insurance type and health insurance premiums; National Health Insurance beneficiaries were divided into quintiles (Q1-Q5) based on their insurance premiums, a proxy for income, and Medical Aid beneficiaries, who consist of around 3% of the total population, were set as the lowest SEP group (Q0). The National Health Insurance Service provides a total of 20 quintiles based on household insurance premiums. We used this information and divided it into 5 bands. It was not possible to adjust for the number of people in the household because data on household size were not provided. In addition, although there is a difference between employment-based policyholders and self-employed subscribers in how insurance premium deciles are calculated, this study did not distinguish between the 2 groups and used the combined premium deciles.

To assess absolute and relative inequality in mortality across the 6 SEP subgroups (Q0-Q5), we calculated the slope index of inequality (SII) and the relative index of inequality (RII) from the years 2017 to 2020. The SII and RII are used to show health inequalities across multiple groups with a hierarchy, such as education or income. The SII shows the magnitude of absolute inequality by calculating the difference in index estimates between the most disadvantaged group and the most advantaged one, taking into account the population size of all subgroups based on a regression model. Meanwhile, the RII shows the extent of relative inequality through the ratio of the most disadvantaged group to the most advantaged one, derived from the same regression model for the SII [22].

Additionally, we examined changes in inequality between health insurance premium deciles (Q1-Q5), excluding Medical Aid beneficiaries (Q0). For absolute inequality, we calculated the rate difference (RD) in mortality between Q1 and Q5, and for relative inequality, we calculated the rate ratio (RR) between Q1 and Q5.

Statistical analyses were performed using R version 4.1.1 (R Foundation for Statistical Computing, Vienna, Austria), and the PHEindicatormethods package was used to calculate the SII and RII [23].

### Ethics statement

The study protocol was approved by the Institutional Review Board of the National Medical Center of Korea (IRB No. NMC-2021-06-078) and the National Health Insurance Service (NHIS-2021-1-850).

## RESULTS

### Overall change in mortality from 2017 to 2020

Annually, the ASMR of all-cause, avoidable, preventable, and treatable deaths decreased from 2017 to 2020. In contrast, the ASMR of unavoidable deaths increased and decreased repeatedly, increasing by 0.97% in 2020 compared with 2019 (Supplementary Material 3).

### Absolute and relative inequalities of mortality from 2017 to 2020

#### All-cause mortality

All-cause mortality rates increased in Q0 (Medical Aid beneficiaries) between 2018 and 2020 and in Q2 between 2019 and 2020. They decreased in all other SEP groups between 2019 and 2020. When analyzed by gender, all-cause mortality increased in 2020 compared to 2019 in Q0 and Q2 for men and in Q2, Q3, and Q4 for women (Table 1).

Both the SII and RII of all-cause mortality increased steadily from 2017 to 2020. The SII and RII increased in men for 4 consecutive years, whereas the SII for women decreased slightly in 2020 compared to 2019, while the RII remained unchanged (Table 1). The RD and RR values comparing Q1 and Q5 increased from 2017 to 2019 and then decreased in 2020.

#### Avoidable and unavoidable mortality

The avoidable mortality rates in Q2 increased slightly between 2019 and 2020, while they continued to decrease in Q3, Q4, and Q5 from 2017 to 2020. When analyzed by gender, the avoidable mortality rates increased for men in Q2 and for women in Q2, Q3, and Q4 compared to 2019. The SII of avoidable mortality showed a slight decrease in 2020 compared to 2019, while the RII increased for 4 consecutive years. The RII increased from 2017 to 2020 for men and increased from 2017 to 2019 for women, before decreasing slightly in 2020. The RD and RR of Q1 and Q5 increased from 2017 to 2019 and then decreased in 2020 (Table 2).

The unavoidable mortality rate of Q0 increased for 4 consecutive years. For men, Q0, Q1, and Q2 showed an increase in 2020 compared to 2019. For women, those in Q0, Q2, Q3, and Q4 showed an increase in 2020 compared to 2019. Both the SII and RII increased for the unavoidable mortality rate in 2020 compared to 2019. The RD and RR values comparing the Q1 and Q5 also increased for both men and women in 2020 compared to 2019 (Table 3).

#### Treatable and preventable mortality

Although there was an increase in the treatable and preventable mortality rates in Q2 in 2020 compared to 2019, the rest of the groups showed a decline in the mortality rate. There was an increase in treatable mortality rate in Q2 for men, but no group showed an increase in mortality for women in 2020. There was an increase in the preventable mortality rate in Q2 for men and in Q2, Q3, and Q4 for women in 2020 compared to 2019.

The SII and RII for treatable mortality decreased slightly in 2020 compared to 2019 (Table 4). The RD and RR of Q1 and Q5 also decreased marginally in 2020. However, the magnitude of both absolute and relative inequality increased in 2020 compared to 2019 for preventable mortality (Table 5). The RD and RR values decreased in 2020 for preventable mortality in the entire population, but the RR values for women increased in 2020.

## DISCUSSION

**Figure 1.**
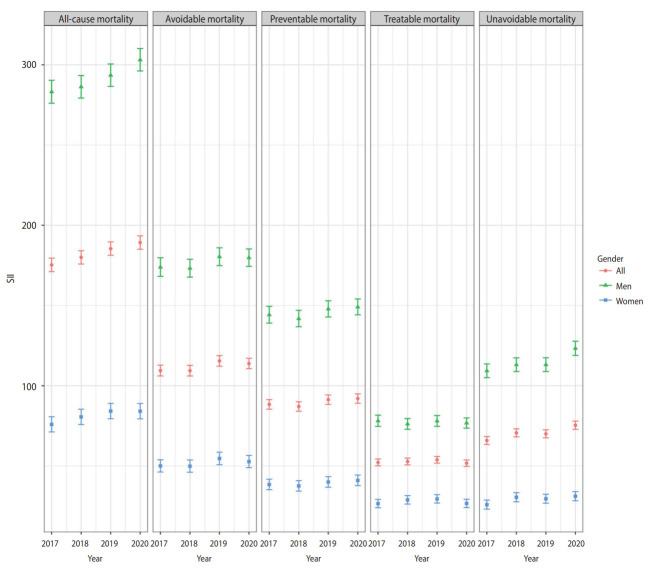
Slope index of inequalities (SIIs) from 2017 to 2020.

**Figure 2.**
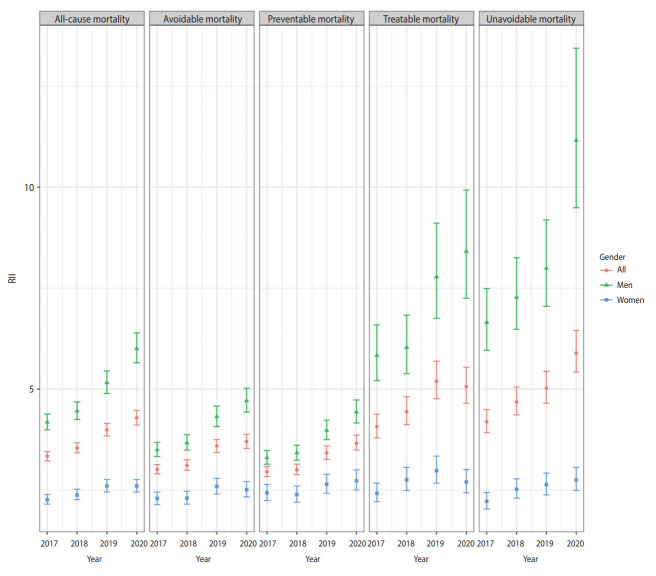
Relative index of inequalities (RIIs) from 2017 to 2020.

The ASMRs for all-cause, avoidable, treatable, and preventable deaths from 2017 to 2020 showed a continuous decline. In contrast, for non-avoidable deaths, the ASMR fluctuated and showed almost no change. This result is consistent with a study indicating that Korea’s unavoidable mortality rate leveled off after 2003 and 2004 [24]. The disparity in all-cause mortality between income classes has widened. Specifically, the preventable and unavoidable mortality rates exhibited an apparent increase in inequality, as both the SII and RII rose in 2020. Unavoidable mortality was an exception in that not only did inequality increase, but outcomes for the entire population showed no improvement (Figures 1 and 2).

It is a favorable finding that the improvement in all-cause mortality is mostly attributable to a decline in avoidable deaths. In addition, the continuously declining pattern of treatable mortality in Korea, despite the impact of COVID-19, suggests that the healthcare system was quite stable during the first year of the COVID-19 pandemic. In Korea, the number of COVID-19 patients in the first year of the pandemic was relatively small. The cumulative number of confirmed COVID-19 cases in Korea was 61,769 as of December 31, 2020, which was much lower than in the United States (20,219,871), the United Kingdom (2,488,780), and Italy (2,107,166) [25]. Thus, in contrast to Western societies, Korea seems to have prevented the collapse of the healthcare system. This also seems to explain the results of previous studies on excess death, which showed no substantial increase in mortality in 2020 [6-10].

Meanwhile, although the declining pattern was not halted during COVID-19, special attention must be paid to preventable mortality. Preventable deaths account for a large proportion of avoidable deaths and showed a wider gender gap during COVID-19. Not only Q2, but Q3 and Q4, also showed an increase in preventable mortality in women. In addition, absolute and relative inequalities in preventable mortality increased during the pandemic. The increase in preventable mortality and related inequalities suggests a potential failure of public health interventions focused on the broader social determinants of health. As the pandemic continues and socioeconomic inequalities deepen [26], it is important to continue monitoring whether inequalities in preventable mortality increase.

Among the categories of causes of death, cancer, injuries, and circulatory diseases accounted for 82.16% of all avoidable deaths in 2020 (Supplementary Material 4). We further examined changes in mortality inequality for avoidable deaths from these major 3 causes of death (Supplementary Materials 5-7). The inequality in death from injuries was largely responsible for the magnitude of absolute inequality in avoidable mortality. Suicide accounted for the largest proportion of deaths from injury. For men, the SII of suicide decreased slightly from 2019 to 2020, but for women, both the SII and RII of suicide increased slightly from 2019 to 2020 (Supplementary Material 8). Thus, it is worth keeping an eye on whether inequalities in suicide rates for women have increased, and further research extending beyond the first year of the COVID-19 pandemic is needed.

Moreover, it is noteworthy that absolute and relative inequality worsened for both men and women in unavoidable mortality. Unlike avoidable mortality, unavoidable mortality did not show a decreasing pattern in the entire population. This raises a grave concern because increasing inequality is combined with stagnating outcomes for the entire population. Benach et al. [27] summarized the policy implications of the various possible scenarios depending on the combination of changes (maintenance, deterioration, and improvement) in the health level and inequality level of the entire population. The unavoidable deaths in our study reflect a scenario in which health outcomes are stable at the overall population level, but inequality increases. This reflects a situation where society’s inability to provide adequate support to those who need help the most harms the entire level of health outcome [27], suggesting that the problem of unfair distribution also hinders the improvement of outcomes in the entire population.

Conceptually, unavoidable mortality refers to an area beyond human control and cannot be solved by mobilizing given resources and current technologies. Thus, there are some views that healthcare interventions are impossible and measurements of inequality are not suitable for unavoidable death [28]. From the standpoint of healthcare, socioeconomic inequality in unavoidable death should be minimal since healthcare cannot make any difference in the area. However, our observation, similar to a prior study that identified a clear inverse relationship between SES and unavoidable death [29], suggests that socioeconomic inequality in this area is not trivial, and there may still be some amenable factors generated by social determinants of health.

Several aspects of unavoidable causes of death can support this view. First of all, there is no clear boundary between “avoidable” and “unavoidable,” and there are few causes of death that are entirely “avoidable” or “unavoidable” [30,31]. For example, rare cancers experienced by a small number of people such as C23-C26 (biliary tract and pancreatic family), C30-C32 (nasal and laryngeal cancer), and C37-C39 (mediastinal sinus cancer) belong to unavoidable causes of death. However, although a complete cure is impossible, there is still a chance to delay death through early diagnosis and treatment.

Furthermore, unavoidable causes of death include the ICD-10 codes of R00-R99 (symptoms, signs and abnormal clinical and laboratory findings, not elsewhere classified). This cause of death has increased [9] and showed substantial excess death (2,756; 95% CI, 2,021 to 3,378) [10] in Korea during the pandemic. Furthermore, a study examining the mortality pattern of the different causes of death before and during the COVID-19 pandemic using an interrupted time series analysis showed that the deaths observed outside medical facilities increased, and this increase was mainly attributable to neoplasms and unclassified causes of death [32]. Thus, it is highly convincing to assume that deaths for which the specific cause was not given could have been poorly diagnosed and treated due to the affected individuals’ low socioeconomic status. Thus, the increase in inequality in the unavoidable death seems “avoidable” if socioeconomic conditions are well addressed.

Korea’s mortality rate from R00-R99 was the highest among OECD countries after Poland in 2019 [33]. The magnitude of R-code deaths can lead to an underestimation of the size of avoidable deaths, as deaths with R-code could have been classified as avoidable if they were properly classified [34]. Since the high proportion of R-codes in Korea highlights a problem with the quality of Korea’s cause-of-death statistics [35], there is a need to expand efforts to reduce unexplained deaths by systematically analyzing cases where the causes of death were unknown. Meanwhile, the increase in R-coded deaths during COVID-19 can be attributed to out-of-hospital deaths of vulnerable people who were unable to access public hospitals [36,37] and unattended deaths of people who lacked social networks. In this study, we could not analyze unavoidable deaths in detail due to data availability, but future studies should consider exploring them.

This study has the following limitations. First, we only covered the first year of the COVID-19 pandemic. In particular, Korea did not have a large outbreak in the first year. Thus, the study period may have been too short to determine the impact of COVID-19. However, we could still confirm the pattern of exacerbated inequality despite the overall mortality decrease. This suggests a strong need for follow-up studies.

In addition, as we were unable to use a detailed classification of certain causes of death for analysis due to privacy purposes, we incorporated these causes into a broader category. Thus, the ICD-10 codes corresponding to each disease group shown in Supplementary Material 2 may be slightly inaccurate. For example, the ICD-10 code of malaria is B50-B54, but we had no choice but to use B50-B64, representing all protozoal diseases. However, deaths other than malaria are negligible and would not have made a substantial difference. There were no deaths from B55 (leishmaniasis) or B56-B57 (trypanosomiasis) during 2017-2020, according to Statistics Korea [38].

Lastly, the type of healthcare coverage or the health insurance premium quintile used in this study as a proxy for SEP may not have accurately reflected the income class. However, these indicators have shown consistent results with various health outcome indicators in previous studies. In addition, we considered the distribution of the entire income group by using a regression-based scale such as the SII and RII, rather than a paired comparison of the highest and lowest groups. Although the mortality rate of the entire population declined, the annual mortality rate of the middle class, such as Q2, Q3, and Q4, has not decreased consistently in specific groups defined according to gender and causes of death. These results would never have been identified if only the highest and lowest pairs were compared.

The results of this study can be summarized as follows. The mortality inequality increased in the first year of COVID-19, even though there were no signs of a worsening trend in mortality inequality. We must continue to evaluate mortality disparities, especially regarding unavoidable and preventable mortality.

## Figures and Tables

**Figure f3-epih-45-e2023072:**
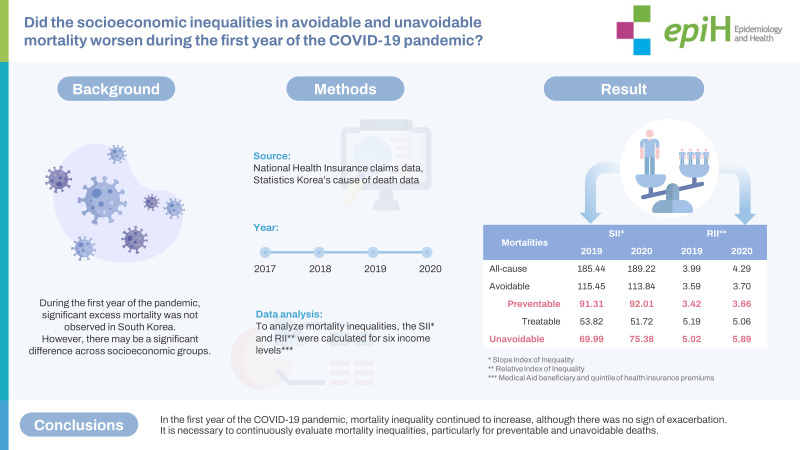


**Table 1.** Annual ASMRs and absolute and relative inequality for all-cause mortalities from 2017 to 2020

Variables	All				Men				Women			
	2017	2018	2019	2020	2017	2018	2019	2020	2017	2018	2019	2020
Q0	822.72 (805.43, 840.21)	819.59 (802.75, 836.61)	840.83 (822.80, 859.06)	848.30 (830.18, 866.62)	1198.17 (116.96, 1,228.79)	1,172.69 (1,143.95, 1,201.81)	1,185.22 (1,154.51, 1,216.32)	1,199.73 (1,169.05, 1,230.79)	468.76 (449.67, 488.26)	477.23 (458.07, 496.80)	496.92 (476.44, 517.82)	491.36 (470.66, 512.51)
Q1	168.62 (165.93, 171.35)	167.58 (164.97, 170.22)	175.71 (173.17, 178.28)	167.95 (165.41, 170.51)	269.03 (264.02, 274.11)	262.41 (257.61, 267.27)	268.84 (264.30, 273.43)	259.34 (254.77, 263.96)	88.83 (86.09, 91.64)	90.75 (88.06, 93.50)	96.76 (94.08, 99.49)	93.35 (90.65, 96.11)
Q2	166.47 (163.73, 169.24)	166.49 (163.74, 169.28)	147.49 (144.81, 150.20)	155.01 (152.36, 157.70)	241.87 (237.24, 246.55)	243.48 (238.79, 248.23)	219.04 (214.38, 223.78)	229.01 (224.40, 233.68)	92.78 (89.76, 95.88)	91.99 (88.99, 95.05)	81.44 (78.54, 84.42)	86.16 (83.28, 89.10)
Q3	163.63 (161.16, 166.14)	160.31 (157.88, 162.77)	152.25 (149.90, 154.62)	149.73 (147.44, 152.05)	228.62 (224.52, 232.77)	220.23 (216.26, 224.25)	211.90 (208.05, 215.81)	204.97 (201.23, 208.76)	96.98 (94.25, 99.77)	98.80 (96.03, 101.62)	91.06 (88.44, 93.73)	93.84 (91.21, 96.51)
Q4	143.04 (141.04, 145.06)	140.34 (138.37, 142.33)	130.91 (129.03, 132.81)	126.31 (124.46, 128.18)	195.29 (192.03, 198.59)	189.59 (186.41, 192.81)	178.48 (175.44, 181.56)	167.85 (164.92, 170.83)	91.33 (89.01, 93.69)	91.24 (88.91, 93.61)	83.31 (81.10, 85.56)	84.58 (82.33, 86.87)
Q5	112.00 (110.36, 113.65)	109.09 (107.46, 110.73)	104.03 (102.45, 105.63)	100.23 (98.67, 101.81)	152.73 (150.02, 155.46)	147.79 (145.13, 150.49)	138.13 (135.59, 140.69)	132.40 (129.92, 134.90)	76.55 (74.59, 78.55)	75.51 (73.53, 77.52)	74.34 (72.37, 76.35)	72.11 (70.14, 74.11)
Inequality												
SII	175.31 (171.14, 179.54)	179.99 (175.85, 184.18)	185.44 (181.33, 189.67)	189.22 (185.08, 193.42)	283.20 (276.08, 290.39)	286.28 (279.30, 293.31)	293.49 (286.55, 300.53)	303.08 (296.17, 310.16)	75.89 (71.21, 80.67)	80.56 (75.83, 85.37)	84.20 (79.44, 89.00)	84.09 (79.37, 88.94)
RII	3.34 (3.22, 3.45)	3.54 (3.42, 3.67)	3.99 (3.84, 4.15)	4.29 (4.11, 4.47)	4.18 (3.99, 4.38)	4.46 (4.25, 4.68)	5.16 (4.89, 5.45)	6.00 (5.65, 6.39)	2.26 (2.15, 2.39)	2.38 (2.26, 2.52)	2.60 (2.45, 2.76)	2.60 (2.45, 2.76)
RD (Q1-Q5)	56.62	58.49	71.68	67.72	116.3	114.62	130.71	126.94	12.28	15.24	22.42	21.24
RR (Q1/Q5)	1.51	1.54	1.69	1.68	1.76	1.78	1.95	1.96	1.16	1.20	1.30	1.29

Values of Q0-Q5 are presented as ASMR per 100,000 population (95% confidence interval). ASMR, age-standardized mortality rate; SII, slope index of inequality; RII, relative index of inequality; RD, rate difference; RR, rate ratio; Q0, Medicaid beneficiaries; Q1-Q5, quintile of national health insurance premiums.

**Table 2.** Annual ASMRs and absolute and relative inequality for avoidable mortalities from 2017 to 2020

Variables	All				Men				Women			
	2017	2018	2019	2020	2017	2018	2019	2020	2017	2018	2019	2020
Q0	506.38 (493.01, 519.94)	485.56 (472.88, 498.42)	504.05 (490.35, 517.94)	488.26 (474.93, 501.79)	732.28 (709.33, 755.61)	686.42 (665.12, 708.09)	696.42 (673.80, 719.42)	675.11 (653.27, 697.30)	294.65 (279.49, 310.22)	290.45 (275.71, 305.58)	310.56 (294.30, 327.26)	296.29 (280.27, 312.75)
Q1	113.26 (111.04, 115.50)	112.11 (109.97, 114.28)	117.23 (115.15, 119.34)	109.73 (107.67, 111.81)	181.19 (177.06, 185.38)	176.95 (173.00, 180.95)	180.50 (176.78, 184.29)	169.33 (165.63, 173.09)	58.90 (56.67, 61.19)	59.36 (57.16, 61.61)	63.62 (61.44, 65.86)	60.74 (58.55, 62.98)
Q2	112.54 (110.29, 114.83)	110.98 (108.73, 113.26)	100.06 (97.85, 102.30)	103.63 (101.47, 105.83)	163.60 (159.80, 167.46)	163.21 (159.37, 167.12)	147.90 (144.07, 151.79)	151.76 (148.02, 155.56)	61.80 (59.35, 64.32)	59.87 (57.46, 62.34)	55.23 (52.83, 57.70)	58.12 (55.76, 60.55)
Q3	110.60 (108.56, 112.66)	107.52 (105.53, 109.55)	102.25 (100.32, 104.20)	101.28 (99.39, 103.20)	156.55 (153.15, 160.00)	150.67 (147.39, 154.01)	143.43 (140.26, 146.66)	140.12 (137.02, 143.26)	63.49 (61.28, 65.76)	63.12 (60.91, 65.38)	59.93 (57.78, 62.13)	61.82 (59.67, 64.02)
Q4	98.44 (96.77, 100.13)	94.78 (93.15, 96.43)	87.30 (85.75, 88.86)	82.17 (80.67, 83.70)	137.90 (135.16, 140.69)	130.61 (127.96, 133.30)	120.70 (118.20, 123.25)	110.18 (107.80, 112.61)	59.38 (57.49, 61.31)	58.88 (56.99, 60.81)	53.85 (52.05, 55.70)	54.28 (52.44, 56.16)
Q5	76.02 (74.65, 77.40)	73.69 (72.33, 75.06)	69.96 (68.65, 71.30)	66.90 (65.60, 68.22)	106.62 (104.35, 108.93)	102.19 (99.95, 104.45)	94.69 (92.57, 96.84)	89.74 (87.68, 91.84)	49.28 (47.68, 50.91)	48.85 (47.22, 50.52)	48.31 (46.68, 49.97)	46.74 (45.11, 48.40)
Inequality												
SII	109.45 (106.09, 112.85)	109.39 (106.08, 112.71)	115.45 (112.15, 118.79)	113.84 (110.61, 117.12)	173.92 (168.16, 179.75)	173.18 (167.68, 178.83)	180.39 (174.89, 185.97)	179.80 (174.41, 185.26)	50.02 (46.22, 53.87)	49.87 (46.08, 53.75)	54.66 (50.78, 58.59)	52.71 (48.92, 56.61)
RII	3.01 (2.90, 3.13)	3.11 (2.99, 3.25)	3.59 (3.43, 3.75)	3.70 (3.53, 3.88)	3.50 (3.33, 3.68)	3.67 (3.49, 3.87)	4.32 (4.07, 4.58)	4.71 (4.43, 5.02)	2.29 (2.14, 2.45)	2.30 (2.15, 2.47)	2.59 (2.40, 2.79)	2.51 (2.33, 2.71)
RD (Q1-Q5)	37.24	38.42	47.27	42.83	74.57	74.76	85.81	79.59	9.62	10.51	15.31	14.00
RR (Q1/Q5)	1.49	1.52	1.68	1.64	1.70	1.73	1.91	1.89	1.20	1.22	1.32	1.30

Values of Q0-Q5 are presented as ASMR per 100,000 population (95% confidence interval). ASMR, age-standardized mortality rate; SII, slope index of inequality; RII, relative index of inequality; RD, rate difference; RR, rate ratio; Q0, Medicaid beneficiaries; Q1-Q5, quintile of national health insurance premiums.

**Table 3.** Annual ASMRs and absolute and relative inequality for unavoidable mortalities from 2017 to 2020

Variables	All				Men				Women			
	2017	2018	2019	2020	2017	2018	2019	2020	2017	2018	2019	2020
Q0	316.34 (305.41, 327.47)	334.03 (323.00, 345.26)	336.78 (325.09, 348.68)	360.04 (347.81, 372.48)	465.89 (446.33, 485.90)	486.27 (467.05, 505.89)	488.79 (468.12, 509.90)	524.62 (503.15, 546.52)	174.11 (162.60, 186.04)	186.14 (174.02, 198.70)	186.35 (174.01, 199.13)	193.35 (180.42, 206.72)
Q1	55.37 (53.83, 56.93)	55.47 (53.98, 56.99)	58.48 (57.03, 59.96)	58.22 (56.75, 59.72)	87.84 (85.01, 90.75)	85.46 (82.73, 88.25)	88.34 (85.76, 90.97)	90.01 (87.35, 92.73)	29.93 (28.34, 31.58)	31.39 (29.84, 32.99)	33.14 (31.60, 34.73)	32.61 (31.04, 34.24)
Q2	53.93 (52.38, 55.52)	55.52 (53.93, 57.13)	47.43 (45.93, 48.97)	51.38 (49.85, 52.94)	78.27 (75.65, 80.95)	80.27 (77.59, 83.01)	71.15 (68.50, 73.87)	77.25 (74.56, 80.00)	30.98 (29.22, 32.81)	31.82 (30.07, 33.64)	26.22 (24.61, 27.90)	28.04 (26.41, 29.74)
Q3	53.04 (51.64, 54.46)	52.79 (51.40, 54.20)	50.00 (48.67, 51.36)	48.45 (47.17, 49.76)	72.07 (69.79, 74.41)	69.56 (67.34, 71.82)	68.47 (66.29, 70.71)	64.85 (62.76, 66.98)	33.49 (31.90, 35.14)	35.68 (34.03, 37.39)	31.13 (29.64, 32.68)	32.02 (30.52, 33.56)
Q4	44.61 (43.51, 45.72)	45.56 (44.45, 46.69)	43.62 (42.55, 44.70)	44.14 (43.06, 45.23)	57.39 (55.65, 59.17)	58.98 (57.23, 60.77)	57.78 (56.07, 59.53)	57.67 (55.98, 59.41)	31.95 (30.62, 33.33)	32.36 (31.00, 33.76)	29.46 (28.19, 30.76)	30.30 (29.01, 31.63)
Q5	35.98 (35.08, 36.90)	35.40 (34.50, 36.31)	34.06 (33.19, 34.95)	33.34 (32.48, 34.21)	46.10 (44.65, 47.59)	45.60 (44.16, 47.08)	43.44 (42.05, 44.86)	42.65 (41.30, 44.03)	27.28 (26.14, 28.45)	26.66 (25.54, 27.81)	26.03 (24.92, 27.17)	25.37 (24.27, 26.49)
inequality												
SII	65.87 (63.42, 68.38)	70.60 (68.12, 73.13)	69.99 (67.50, 72.53)	75.38 (72.84, 77.96)	109.28 (105.06, 113.60)	113.10 (108.88, 117.38)	113.11 (108.87, 117.45)	123.28 (118.89, 127.74)	25.87 (23.09, 28.74)	30.46 (27.66, 33.37)	29.54 (26.78, 32.38)	31.13 (28.30, 34.05)
RII	4.19 (3.92, 4.49)	4.68 (4.36, 5.05)	5.02 (4.65, 5.44)	5.89 (5.42, 6.45)	6.65 (5.96, 7.49)	7.27 (6.48, 8.25)	7.99 (7.05, 9.19)	11.16 (9.49, 13.44)	2.22 (2.03, 2.44)	2.52 (2.30, 2.78)	2.63 (2.38, 2.92)	2.75 (2.49, 3.06)
RD (Q1-Q5)	19.39	20.07	24.42	24.88	41.74	39.86	44.90	47.36	2.65	4.73	7.11	7.24
RR (Q1/Q5)	1.54	1.57	1.72	1.75	1.91	1.87	2.03	2.11	1.10	1.18	1.27	1.29

Values of Q0-Q5 are presented as ASMR per 100,000 population (95% confidence interval). ASMR, age-standardized mortality rate; SII, slope index of inequality; RII, relative index of inequality; RD, rate difference; RR, rate ratio; Q0, Medicaid beneficiaries; Q1-Q5, quintile of national health insurance premiums.

**Table 4.** ASMRs and absolute and relative inequality for treatable mortalities from 2017 to 2020

Variables	All				Men				Women			
	2017	2018	2019	2020	2017	2018	2019	2020	2017	2018	2019	2020
Q0	262.82 (253.63, 272.19)	262.64 (253.74, 271.70)	266.93 (257.27, 276.77)	253.36 (244.40, 262.48)	364.88 (349.20, 380.94)	351.43 (337.26, 365.91)	352.58 (336.73, 368.80)	337.31 (323.15, 351.79)	167.38 (156.88, 178.25)	175.26 (164.17, 186.74)	181.09 (169.49, 193.08)	166.01 (154.77, 177.65)
Q1	43.97 (42.62, 45.35)	42.26 (40.98, 43.56)	42.18 (40.97, 43.40)	40.06 (38.86, 41.28)	64.15 (61.76, 66.61)	60.16 (57.94, 62.44)	59.14 (57.08, 61.25)	55.63 (53.60, 57.72)	27.76 (26.28, 29.29)	27.59 (26.16, 29.07)	27.80 (26.42, 29.23)	27.30 (25.90, 28.76)
Q2	41.32 (39.97, 42.70)	39.73 (38.41, 41.09)	37.27 (35.95, 38.63)	37.52 (36.25, 38.82)	52.81 (50.70, 54.98)	52.67 (50.54, 54.87)	49.14 (46.99, 51.36)	49.98 (47.92, 52.10)	29.65 (27.98, 31.39)	26.83 (25.25, 28.48)	25.86 (24.26, 27.54)	25.46 (23.95, 27.05)
Q3	41.76 (40.52, 43.03)	40.07 (38.87, 41.30)	38.37 (37.20, 39.55)	38.16 (37.02, 39.32)	52.16 (50.21, 54.17)	48.83 (46.97, 50.73)	47.80 (45.99, 49.66)	47.54 (45.76, 49.37)	31.46 (29.93, 33.05)	31.30 (29.78, 32.88)	28.89 (27.44, 30.39)	28.76 (27.35, 30.22)
Q4	37.58 (36.58, 38.61)	35.88 (34.91, 36.87)	32.44 (31.53, 33.37)	31.36 (30.46, 32.27)	45.78 (44.23, 47.37)	43.89 (42.39, 45.42)	39.47 (38.07, 40.91)	37.08 (35.74, 38.46)	24.21 (23.16, 25.30)	22.78 (21.75, 23.84)	22.18 (21.16, 23.23)	21.93 (20.87, 23.01)
Q5	28.86 (28.06, 29.67)	27.52 (26.75, 28.32)	25.79 (25.05, 26.55)	24.89 (24.15, 25.66)	35.14 (33.88, 36.43)	33.91 (32.69, 35.17)	30.85 (29.72, 32.02)	29.26 (28.15, 30.40)	24.21 (23.16, 25.30)	22.78 (21.75, 23.84)	22.18 (21.16, 23.23)	21.93 (20.87, 23.01)
Inequality												
SII	52.19 (50.09, 54.37)	52.76 (50.68, 54.86)	53.82 (51.75, 55.93)	51.72 (49.72, 53.75)	78.13 (74.65, 81.66)	76.15 (72.85, 79.54)	78.02 (74.67, 81.43)	76.72 (73.55, 79.94)	26.52 (23.96, 29.16)	28.85 (26.25, 31.52)	29.45 (26.88, 32.11)	26.64 (24.10, 29.26)
RII	4.07 (3.79, 4.38)	4.44 (4.12, 4.81)	5.19 (4.76, 5.69)	5.06 (4.65, 5.54)	5.83 (5.21, 6.59)	6.03 (5.38, 6.83)	7.78 (6.75, 9.11)	8.41 (7.25, 9.93)	2.42 (2.21, 2.67)	2.75 (2.49, 3.06)	2.98 (2.67, 3.34)	2.70 (2.43, 3.01)
RD (Q1-Q5)	15.11	14.74	16.39	15.17	29.01	26.25	28.29	26.37	3.55	4.81	5.62	5.37
RR (Q1/Q5)	1.52	1.54	1.64	1.61	1.83	1.77	1.92	1.90	1.15	1.21	1.25	1.24

Values of Q0-Q5 are presented as ASMR per 100,000 population (95% confidence interval). ASMR, age-standardized mortality rate; SII, slope index of inequality; RII, relative index of inequality; RD, rate difference; RR, rate ratio; Q0, Medicaid beneficiaries; Q1-Q5, quintile of national health insurance premiums.

**Table 5.** Annual ASMRs and absolute and relative inequality for preventable mortalities from 2017 to 2020

Variables	All				Men				Women			
	2017	2018	2019	2020	2017	2018	2019	2020	2017	2018	2019	2020
Q0	383.19 (371.66, 394.92)	359.73 (348.84, 370.80)	370.67 (359.20, 382.33)	362.85 (351.33, 374.55)	571.94 (551.97, 592.29)	526.68 (508.15, 545.56)	532.15 (512.95, 551.71)	517.83 (498.65, 537.37)	204.68 (191.86, 217.93)	196.46 (184.12, 209.21)	207.48 (194.15, 221.25)	202.88 (189.50, 216.70)
Q1	94.19 (92.18, 96.23)	93.48 (91.53, 95.46)	98.29 (96.39, 100.22)	92.10 (90.22, 94.01)	156.74 (152.91, 160.63)	153.83 (150.14, 157.58)	157.30 (153.82, 160.84)	148.19 (144.72, 151.72)	44.40 (42.47, 46.39)	44.60 (42.71, 46.55)	48.34 (46.44, 50.31)	46.06 (44.16, 48.00)
Q2	95.47 (93.41, 97.57)	94.1 (92.04, 96.19)	83.45 (81.43, 85.50)	87.30 (85.31, 89.32)	145.33 (141.75, 148.97)	143.65 (140.06, 147.30)	129.51 (125.92, 133.17)	133.63 (130.12, 137.22)	46.16 (44.05, 48.34)	45.88 (43.78, 48.05)	40.69 (38.63, 42.83)	43.88 (41.83, 46.00)
Q3	92.33 (90.47, 94.22)	88.89 (87.08, 90.73)	83.84 (82.10, 85.61)	82.64 (80.93, 84.37)	136.99 (133.82, 140.21)	130.33 (127.28, 133.44)	123.71 (120.77, 126.71)	120.06 (117.20, 122.97)	46.26 (44.37, 48.21)	46.09 (44.20, 48.05)	42.73 (40.91, 44.60)	44.61 (42.77, 46.50)
Q4	80.93 (79.41, 82.47)	77.67 (76.18, 79.17)	71.52 (70.11, 72.95)	66.78 (65.41, 68.17)	119.50 (116.94, 122.10)	112.93 (110.47, 115.44)	104.31 (101.97, 106.68)	94.63 (92.41, 96.89)	42.17 (40.57, 43.81)	41.69 (40.09, 43.33)	38.04 (36.50, 39.61)	38.31 (36.75, 39.92)
Q5	61.75 (60.51, 63.00)	59.99 (58.75, 61.24)	56.94 (55.74, 58.15)	53.75 (52.58, 54.94)	91.69 (89.57, 93.84)	87.98 (85.90, 90.09)	81.42 (79.44, 83.42)	76.70 (74.78, 78.65)	34.71 (33.36, 36.09)	34.73 (33.33, 36.16)	34.62 (33.22, 36.05)	32.46 (31.11, 33.86)
Inequality												
SII	88.35 (85.38, 91.38)	87.05 (84.11, 90.03)	91.31 (88.38, 94.26)	92.01 (89.11, 94.93)	144.22 (139.03, 149.47)	141.82 (136.78, 146.98)	147.87 (142.85, 152.93)	149.1 (144.20, 154.10)	38.43 (35.20, 41.74)	37.55 (34.32, 40.85)	40.00 (36.74, 43.32)	40.99 (37.75, 44.33)
RII	2.95 (2.83, 3.08)	3.00 (2.88, 3.14)	3.42 (3.26, 3.59)	3.66 (3.49, 3.86)	3.30 (3.14, 3.48)	3.42 (3.24, 3.61)	3.98 (3.75, 4.23)	4.43 (4.16, 4.73)	2.43 (2.24, 2.64)	2.39 (2.20, 2.60)	2.64 (2.42, 2.89)	2.73 (2.50, 3.00)
RD (Q1-Q5)	32.44	33.49	41.35	38.35	65.05	65.85	75.88	71.49	9.69	9.87	13.72	13.60
RR (Q1/Q5)	1.53	1.56	1.73	1.71	1.71	1.75	1.93	1.93	1.28	1.28	1.40	1.42

Values of Q0-Q5 are presented as ASMR per 100,000 population (95% confidence interval). ASMR, age-standardized mortality rate; SII, slope index of inequality; RII, relative index of inequality; RD, rate difference; RR, rate ratio; Q0, Medicaid beneficiaries; Q1-Q5, quintile of national health insurance premiums.

## SUPPLEMENTARY MATERIALS

Supplement Material 1. Population and deaths by year, gender, and income quintile

Supplement Material 2. List of avoidable, treatable, and preventable deaths used in this study

Supplement Material 3. Annual ASMRs from 2017 to 2020 (unit: per 100,000 people)

Supplement Material 4. Annual ASMRs from avoidable causes (cancer, injuries, circulatory) from 2017 to 2020 (unit: per 100,000 people)

Supplement Material 5. Annual ASMRs and absolute and relative inequality of ASMRs due to cancer from 2017 to 2020

Supplement Material 6. Annual ASMRs and absolute and relative inequality of ASMRs due to injuries from 2017 to 2020

Supplement Material 7. Annual ASMRs and absolute and relative inequality of ASMRs due to diseases of the circulatory system from 2017 to 2020

Supplement Material 8. Annual ASMRs and absolute and relative inequality for suicides from 2017 to 2020
