# Supplementary material for: Did the socioeconomic inequalities in avoidable and unavoidable mortality worsen during the first year of the COVID-19 pandemic in Korea?
Source: Epidemiol Health. 2023 Aug 3;45:e2023072. doi: 10.4178/epih.e2023072 (PMC10728611; doi:10.4178/epih.e2023072)
Supplement: Supplement Material 1. — Population and deaths by year, gender, and income quintile [file epih-45-e2023072-Supplementary-1.docx]

Supplementary Material 1. Population and deaths by year, gender, and income quintile

|  | Population | Deaths |
| --- | --- | --- |
| Year |  |  |
| 2017 | 51,681,126 | 282,286 |
| 2018 | 51,812,088 | 295,010 |
| 2019 | 52,056,908 | 291,781 |
| 2020 | 52,021,617 | 301,832 |
| Gender |  |  |
| Men | 103,883,325 | 631,987 |
| Women | 103,688,414 | 538,922 |
| Income quintile |  |  |
| Q0 | 5,995,483 | 164,668 |
| Q1 | 32,960,930 | 196,217 |
| Q2 | 29,115,727 | 122,745 |
| Q3 | 36,899,228 | 156,864 |
| Q4 | 45,720,673 | 196,584 |
| Q5 | 56,879,698 | 333,831 |
