# Supplementary material for: Did the socioeconomic inequalities in avoidable and unavoidable mortality worsen during the first year of the COVID-19 pandemic in Korea?
Source: Epidemiol Health. 2023 Aug 3;45:e2023072. doi: 10.4178/epih.e2023072 (PMC10728611; doi:10.4178/epih.e2023072)
Supplement: Supplement Material 2. — List of avoidable, treatable, and preventable deaths used in this study [file epih-45-e2023072-Supplementary-2.docx]

Supplementary Material 2. List of avoidable, treatable, and preventable deaths used in this study

| Disease group | | ICD-10 code | Avoidable | Preventable | Treatable |
| --- | --- | --- | --- | --- | --- |
| Infectious disease | |  |  |  |  |
|  | Conditions that need antibiotic treatment | A30-A49 | ● | ● |  |
|  | COVID-19 etc. | U00-U19 | ● | ● |  |
|  | HIV/AIDS | B20-B24 | ● | ● |  |
|  | Intestinal disease | A00-A09 | ● | ● |  |
|  | Malaria | B50-B64 | ● | ● |  |
|  | Meningitis | G00-G03 | ● | ● |  |
|  | Sexually transmitted infections (except HIV/AIDS) | A50-A64 | ● | ● |  |
|  | Tuberculosis | A15-A19 | ● | ● | ● |
|  | Vaccination | B00-B09 | ● | ● |  |
|  | Viral Hepatitis | B15-B19 | ● | ● |  |
| Cancer | |  |  |  |  |
|  | Benign neoplasm | D10-D36 | ● |  | ● |
|  | Bladder cancer | C67 | ● | ● |  |
|  | Breast cancer (female only) | C50 | ● |  | ● |
|  | Cervical cancer | C53 | ● | ● | ● |
|  | Colorectal cancer | C18-C21 | ● |  | ● |
|  | Hodgkin's disease | C81 | ● |  | ● |
|  | Lip, oral cavity and pharynx cancer | C00-C14 | ● | ● |  |
|  | Liver cancer | C22 | ● | ● |  |
|  | Lung cancer | C33-C34 | ● | ● |  |
|  | Lymphoid leukemia | C91 | ● |  | ● |
|  | Mesothelioma | C45 | ● | ● |  |
|  | Oesophageal cancer | C15 | ● | ● |  |
|  | Skin (melanoma) cancer | C43 | ● | ● |  |
|  | Stomach cancer | C16 | ● | ● |  |
|  | Testicular cancer | C62 | ● |  | ● |
|  | Thyroid cancer | C73 | ● |  | ● |
|  | Uterus cancer | C54, C55 | ● |  | ● |
| Endocrine and metabolic diseases | |  |  |  |  |
|  | Adrenal disorders | E24-E25, E27 | ● |  | ● |
|  | Diabetes mellitus | E10-E14 | ● | ● | ● |
|  | Nutritional deficiency anemia | D50-D53 | ● | ● |  |
|  | Thyroid disorders | E00-E07 | ● |  | ● |
| Diseases of the nervous system | |  |  |  |  |
|  | Epilepsy | G40, G41 | ● |  | ● |
| Diseases of the circulatory system | |  |  |  |  |
|  | Aortic aneurysm | I71 | ● | ● | ● |
|  | Cerebrovascular diseases | I60-I69 | ● | ● | ● |
|  | Hypertensive diseases | I10-I13, I15 | ● | ● | ● |
|  | Ischemic heart diseases | I20-I25 | ● | ● | ● |
|  | Other atherosclerosis | I70, I73 | ● | ● | ● |
|  | Rheumatic and other heart diseases | I00-I09 | ● |  | ● |
|  | Venous thromboembolism | I26, I80, I82 | ● |  | ● |
| Diseases of the respiratory system | |  |  |  |  |
|  | Abscess of lung and mediastinum pyothorax | J85, J86 | ● |  | ● |
|  | Acute lower respiratory infections | J20-J22 | ● |  | ● |
|  | Adult respiratory distress syndrome | J80 | ● |  | ● |
|  | Asthma and bronchiectasis | J45-J47 | ● |  | ● |
|  | Chronic lower respiratory diseases | J40-J44 | ● | ● |  |
|  | Influenza | J09-J11 | ● | ● |  |
|  | Lung diseases due to external agents | J60-J64, J66-J70, J82, J92 | ● | ● |  |
|  | other pleural disorders | J90, J93, J94 | ● |  | ● |
|  | Pneumonia due to streptococcus pneumonia or haemophilus influenza | J13-J14 | ● | ● |  |
|  | Pneumonia, not elsewhere classified or organism unspecified | J12, J15, J16-J18 | ● |  | ● |
|  | Pulmonary edema | J81 | ● |  | ● |
|  | Upper respiratory infections | J00-J06, J30-J39 | ● |  | ● |
| Diseases of the digestive system | |  |  |  |  |
|  | Abdominal hernia | K40-K46 | ● |  | ● |
|  | Acute pancreatitis | K85 | ● |  | ● |
|  | Appendicitis | K35-K38 | ● |  | ● |
|  | Cholelithiasis and cholecystitis | K80-K81 | ● |  | ● |
|  | Gastric and duodenal ulcers | K25-K28 | ● |  | ● |
|  | Other diseases of gallbladder or biliary tract | K82-K83 | ● |  | ● |
|  | Other diseases of pancreas | K86 | ● |  | ● |
| Diseases of the genitourinary system | |  |  |  |  |
|  | Disorders resulting from renal tubular dysfunction | N25 | ● |  | ● |
|  | Inflammatory diseases of genitourinary system | N34, N70-N73, N75, N75, N76 | ● |  | ● |
|  | Nephritis and nephrosis | N00-N07 | ● |  | ● |
|  | Obstructive uropathy | N13, N20-N21, N35 | ● |  | ● |
|  | prostatic hyperplasia | N40 | ● |  | ● |
|  | Renal colic | N23 | ● |  | ● |
|  | Renal failure | N17-N19 | ● |  | ● |
|  | Unspecified contracted kidney, small kidney of unknown cause | N26-N27 | ● |  | ● |
| Pregnancy, childbirth and the perinatal period | |  |  |  |  |
|  | Certain conditions originating in the perinatal period | P00-P96 | ● |  | ● |
|  | Pregnancy, childbirth and the puerperium | O00-O99 | ● |  | ● |
| Congenital malformations | |  |  |  |  |
|  | Certain congenital malformations (neural tube defects) | Q00, Q01, Q05 | ● | ● |  |
|  | Congenital malformations of the circulatory system (heart defects) | Q20-Q28 | ● |  | ● |
| Adverse effects of medical and surgical care | |  |  |  |  |
|  | Drugs, medicaments and biological substances causing adverse effects in therapeutic use | Y40-Y59 | ● |  | ● |
|  | Medical devices associated with adverse incidents in diagnostic and therapeutic use | Y70–Y82 | ● |  | ● |
|  | Misadventures to patients during surgical and medical care | Y60-Y69, Y83-Y84 | ● |  | ● |
| Injuries | |  |  |  |  |
|  | Accidental Injuries | W00-W19  W20-W49  W50-W64  W65-W74  W75-W84  W85-W 99  X00-X09  X10-X19  X20-X29  X30-X39  X40-X49  X50-X57  X58-X59 | ● | ● |  |
|  | Assault | X85-Y09 | ● | ● |  |
|  | Event of undetermined intent | Y10-Y34 | ● | ● |  |
|  | Intentional self-harm | X60-X84 | ● | ● |  |
|  | Transport Accidents | V01-V99 | ● | ● |  |
