# Supplementary material for: Did the socioeconomic inequalities in avoidable and unavoidable mortality worsen during the first year of the COVID-19 pandemic in Korea?
Source: Epidemiol Health. 2023 Aug 3;45:e2023072. doi: 10.4178/epih.e2023072 (PMC10728611; doi:10.4178/epih.e2023072)
Supplement: Supplement Material 3. — Annual ASMRs from 2017 to 2020 (unit: per 100,000 people) [file epih-45-e2023072-Supplementary-3.docx]

Supplementary Material 3. Annual ASMRs from 2017 to 2020 (unit: per 100,000 people)

Values are presented as ASMR per 100,000 population (95% confidence interval).
ASMR, age-standardized mortality rate

|  | | | All | | | | Men | | | | Women | | | |
| --- | --- | --- | --- | --- | --- | --- | --- | --- | --- | --- | --- | --- | --- | --- |
|  | | | 2017 | 2018 | 2019 | 2020 | 2017 | 2018 | 2019 | 2020 | 2017 | 2018 | 2019 | 2020 |
| All-cause mortality | | | 164.24  (163.24-165.25) | 162.79  (161.80-163.79) | 156.63  (155.66-157.60) | 153.86  (152.91-154.82) | 233.09  (231.39-234.79) | 229.66  (228.00-231.33) | 220.52  (218.91-222.13) | 215.36  (213.78-216.94) | 97.90  (96.79-99.02) | 98.14  (97.03-99.26) | 94.56  (93.47-95.66) | 94.12  (93.04-95.22) |
|  | Avoidable mortality | | 110.41  (109.59-111.25) | 107.97  (107.16-108.79) | 103.80  (103.00-104.59) | 100.52  (99.74-101.30) | 158.82  (157.42-160.23) | 154.27  (152.91-155.65) | 147.28  (145.96-148.61) | 141.24  (139.96-142.53) | 63.92  (63.01-64.83) | 63.27  (62.37-64.18) | 61.65  (60.76-62.55) | 61.08  (60.19-61.97) |
|  |  | Treatable mortality | 44.18  (43.67-44.70) | 43.02  (42.52-43.52) | 40.85  (40.37-41.34) | 39.80  (39.33-40.28) | 56.99  (56.16-57.82) | 55.67  (54.86-56.48) | 52.54  (51.77-53.31) | 51.09  (50.35-51.85) | 32.18  (31.55-32.81) | 31.09  (30.48-31.70 | 29.77  (29.17-30.37) | 29.07  (28.49-29.67) |
|  |  | Preventable mortality | 90.56  (89.81-91.31) | 88.11  (87.37-88.85) | 84.44  (83.73-85.17) | 81.55  (80.85-82.26) | 136.38  (135.08-137.69) | 131.71  (130.44-132.98) | 125.55  (124.33-126.78) | 120.00  (118.81-121.19) | 46.29  (45.52-47.07) | 45.77  (45.00-46.55) | 44.38  (43.62-45.16) | 44.09  (43.33-44.86) |
|  | Unavoidable mortality | | 53.83  (53.26-54.40) | 54.82  (54.25-55.39) | 52.83  (52.28-53.39) | 53.34  (52.79-53.90) | 74.27  (73.32-75.22) | 75.39  (74.45-76.34) | 73.24  (72.32-74.16) | 74.11  (73.20-75.03) | 33.98  (33.34-34.64) | 34.83  (34.18-35.49) | 32.91  (32.29-33.55) | 33.01  (32.39-33.65) |
