# Supplementary material for: Did the socioeconomic inequalities in avoidable and unavoidable mortality worsen during the first year of the COVID-19 pandemic in Korea?
Source: Epidemiol Health. 2023 Aug 3;45:e2023072. doi: 10.4178/epih.e2023072 (PMC10728611; doi:10.4178/epih.e2023072)
Supplement: Supplement Material 4. — Annual ASMRs from avoidable causes (cancer, injuries, circulatory) from 2017 to 2020 (unit: per 100,000 people) [file epih-45-e2023072-Supplementary-4.docx]

Supplementary Material 4. Annual ASMRs from avoidable causes (cancer, injuries, circulatory) from 2017 to 2020 (unit: per 100,000 people)

|  | | | All | | | | Men | | | | Women | | | |
| --- | --- | --- | --- | --- | --- | --- | --- | --- | --- | --- | --- | --- | --- | --- |
|  | | | 2017 | 2018 | 2019 | 2020 | 2017 | 2018 | 2019 | 2020 | 2017 | 2018 | 2019 | 2020 |
| Avoidable  (95% CI) | | | 110.41  (109.59-111.25) | 107.97  (107.16-108.79) | 103.80  (103.00-104.59) | 100.52  (99.74-101.30) | 158.82  (157.42-160.23) | 154.27  (152.91-155.65) | 147.28  (145.96-148.61) | 141.24  (139.96-142.53) | 63.92  (63.01-64.83) | 63.27  (62.37-64.18) | 61.65  (60.76-62.55) | 61.08  (60.19-61.97) |
|  | Cancer  (95% CI) | | 40.80  (40.32-41.29) | 37.97  (37.51-38.43) | 37.41  (36.96-37.86) | 35.59  (35.16-36.02) | 57.16  (56.36-57.98) | 52.41  (51.65-53.18) | 51.30  (50.57-52.05) | 48.28  (47.59-48.99) | 25.65  (25.10-26.21) | 24.56  (24.02-25.11) | 24.41  (23.88-24.94) | 23.79  (23.27-24.32) |
|  | Injuries  (95% CI) | | 31.62  (31.15-32.11) | 32.58  (32.10-33.08) | 31.59  (31.11-32.08) | 30.55  (30.07-31.03) | 46.44  (45.64-47.26) | 47.44  (46.63-48.26) | 45.07  (44.28-45.87) | 42.82  (42.05-43.60) | 16.65  (16.15-17.17) | 17.55  (17.03-18.09) | 17.97  (17.43-18.51) | 18.09  (17.55-18.64) |
|  |  | Suicide  (95% CI) | 18.34  (17.97-18.72) | 20.12  (19.73-20.52) | 20.25  (19.86-20.65) | 19.81  (19.42-20.21) | 25.70  (25.09-26.32) | 28.13  (27.50-28.78) | 27.41  (26.79-28.05) | 26.07  (25.46-26.70) | 10.86  (10.45-11.29) | 11.97  (11.53-12.43) | 12.98  (12.51-13.45) | 13.40  (12.93-13.89) |
|  | Circulatory  (95% CI) | | 18.85  (18.52-19.19) | 17.95  (17.63-18.27) | 16.87  (16.57-17.18) | 16.45  (16.15-16.75) | 27.55  (26.98-28.13) | 26.46  (25.91-27.01) | 24.95  (24.43-25.49) | 24.34  (23.82-24.86) | 10.51  (10.17-10.87) | 9.73  (9.40-10.06) | 9.02  (8.70-9.34) | 8.75  (8.45-9.07) |

Values are presented as ASMR per 100,000 population (95% confidence interval).
ASMR, age-standardized mortality rate
