# Supplementary material for: Did the socioeconomic inequalities in avoidable and unavoidable mortality worsen during the first year of the COVID-19 pandemic in Korea?
Source: Epidemiol Health. 2023 Aug 3;45:e2023072. doi: 10.4178/epih.e2023072 (PMC10728611; doi:10.4178/epih.e2023072)
Supplement: Supplement Material 5. — Annual ASMRs and absolute and relative inequality of ASMRs due to cancer from 2017 to 2020 [file epih-45-e2023072-Supplementary-5.docx]

|  | | All | | | | Men | | | | Women | | | |
| --- | --- | --- | --- | --- | --- | --- | --- | --- | --- | --- | --- | --- | --- |
|  |  | 2017 | 2018 | 2019 | 2020 | 2017 | 2018 | 2019 | 2020 | 2017 | 2018 | 2019 | 2020 |
| ASMR (/100,000) | |  |  |  |  |  |  |  |  |  |  |  |  |
|  | Q0 | 145.00  (138.75-151.40) | 131.94  (125.94-138.10) | 147.44  (140.71-154.33) | 135.61  (129.25-142.14) | 200.13  (189.80-210.77) | 170.80  (161.91-179.97) | 188.01  (178.03-198.29) | 178.16  (168.21-188.40) | 92.93  (85.50-100.71) | 92.96  (84.98-101.32) | 104.16  (95.32-113.41) | 90.84  (82.82-99.25) |
|  | Q1 | 37.18  (36.00-38.38) | 36.30  (35.18-37.45) | 38.47  (37.37-39.58) | 34.74  (33.70-35.81) | 58.25  (56.10-60.46) | 56.16  (54.13-58.25) | 56.83  (54.90-58.80) | 52.53  (50.68-54.43) | 20.05  (18.85-21.31) | 19.75  (18.59-20.96) | 22.80  (21.62-24.03) | 19.91  (18.79-21.08) |
|  | Q2 | 39.68  (38.41-40.98) | 36.72  (35.50-37.97) | 35.36  (34.14-36.61) | 35.67  (34.50-36.86) | 55.36  (53.29-57.50) | 51.90  (49.89-53.97) | 50.82  (48.76-52.94) | 49.53  (47.60-51.52) | 23.03  (21.62-24.51) | 20.61  (19.29-21.99) | 19.77  (18.45-21.16) | 21.37  (20.06-22.74) |
|  | Q3 | 42.44  (41.21-43.69) | 39.87  (38.70-41.07) | 38.36  (37.23-39.52) | 37.91  (36.81-39.03) | 58.68  (56.64-60.77) | 53.71  (51.80-55.67) | 51.67  (49.84-53.55) | 50.59  (48.82-52.40) | 26.35  (24.97-27.78) | 26.13  (24.76-27.55) | 24.80  (23.48-26.16) | 25.23  (23.93-26.57) |
|  | Q4 | 40.09  (39.06-41.13) | 37.01  (36.04-38.00) | 34.69  (33.76-35.63) | 32.75  (31.86-33.66) | 54.68  (53.00-56.39) | 49.10  (47.54-50.70) | 46.52  (45.03-48.04) | 42.39  (40.99-43.83) | 26.70  (25.47-27.98) | 26.07  (24.85-27.32) | 23.94  (22.80-25.12) | 24.32  (23.15-25.53) |
|  | Q5 | 31.77  (30.95-32.61) | 28.62  (27.85-29.40) | 27.56  (26.82-28.32) | 26.23  (25.50-26.97) | 43.47  (42.10-44.87) | 38.58  (37.31-39.87) | 36.35  (35.15-37.57) | 33.83  (32.70-34.98) | 22.87  (21.84-23.94) | 21.17  (20.20-22.19) | 21.03  (20.05-22.03) | 20.97  (19.96-22.01) |
| Inequality | |  |  |  |  |  |  |  |  |  |  |  |  |
| SII | | 24.20  (22.39-26.02) | 24.83  (23.09-26.59) | 29.37  (27.63-31.15) | 26.97  (25.30-28.66) | 39.31  (36.28-42.38) | 39.13  (36.32-41.96) | 44.47  (41.65-47.32) | 43.67  (40.95-46.42) | 7.82  (5.69-10.03) | 8.78  (6.67-10.97) | 12.80  (10.62-15.03) | 8.79  (6.69-10.93) |
| RII | | 1.85  (1.77-1.95) | 1.99  (1.89-2.09) | 2.32  (2.20-2.45) | 2.24  (2.12-2.37) | 2.07  (1.95-2.20) | 2.23  (2.09-2.37) | 2.59  (2.42-2.77) | 2.71  (2.53-2.91) | 1.36  (1.25-1.48) | 1.43  (1.31-1.57) | 1.71  (1.56-1.88) | 1.45  (1.32-1.59) |
| RD (Q1-Q5) | | 5.41 | 7.68 | 10.91 | 8.51 | 14.78 | 17.58 | 20.48 | 18.70 | -2.82 | -1.42 | 1.77 | -1.06 |
| RR (Q1/Q5) | | 1.17 | 1.27 | 1.40 | 1.32 | 1.34 | 1.46 | 1.56 | 1.55 | 0.88 | 0.93 | 1.08 | 0.95 |

Supplementary Material 5. Annual ASMRs and absolute and relative inequality of ASMRs due to cancer from 2017 to 2020

Values of Q0-Q5 are presented as ASMR per 100,000 population (95% confidence interval).
ASMR, age-standardized mortality rate; SII, slope index of inequality; RII, relative index of inequality; RD, rate difference; RR, rate ratio; Q0, Medicaid beneficiaries; Q1-Q5, quintile of national health insurance premiums
