# Supplementary material for: Did the socioeconomic inequalities in avoidable and unavoidable mortality worsen during the first year of the COVID-19 pandemic in Korea?
Source: Epidemiol Health. 2023 Aug 3;45:e2023072. doi: 10.4178/epih.e2023072 (PMC10728611; doi:10.4178/epih.e2023072)
Supplement: Supplement Material 6. — Annual ASMRs and absolute and relative inequality of ASMRs due to injuries from 2017 to 2020 [file epih-45-e2023072-Supplementary-6.docx]

Supplementary Material 6. Annual ASMRs and absolute and relative inequality of ASMRs due to injuries from 2017 to 2020

|  | | All | | | | Men | | | | Women | | | |
| --- | --- | --- | --- | --- | --- | --- | --- | --- | --- | --- | --- | --- | --- |
|  |  | 2017 | 2018 | 2019 | 2020 | 2017 | 2018 | 2019 | 2020 | 2017 | 2018 | 2019 | 2020 |
| ASMR (/100,000) | |  |  |  |  |  |  |  |  |  |  |  |  |
|  | Q0 | 110.43  (102.89-118.25) | 103.15  (96.08-110.49) | 109.71  (101.98-117.72) | 105.32  (97.68-113.25) | 154.16  (141.74-167.12) | 147.12  (134.81-159.96) | 145.89  (133.45-158.86) | 140.05  (127.66-152.97) | 69.14  (60.13-78.81) | 61.27  (53.54-69.57) | 73.89  (64.40-84.07) | 71.14  (61.77-81.18) |
|  | Q1 | 37.57  (36.19-38.99) | 38.38  (37.02-39.78) | 42.65  (41.29-44.04) | 39.98  (38.62-41.38) | 61.32  (58.72-63.99) | 62.36  (59.81-64.98) | 66.59  (64.14-69.10) | 62.52  (60.05-65.06) | 18.83  (17.46-20.26) | 19.30  (17.93-20.74) | 22.42  (21.00-23.91) | 21.56  (20.14-23.06) |
|  | Q2 | 37.39  (36.04-38.78) | 39.14  (37.74-40.58) | 33.08  (31.73-34.48) | 35.80  (34.42-37.21) | 57.26  (54.89-59.71) | 59.98  (57.51-62.53) | 50.37  (47.95-52.86) | 54.06  (51.62-56.58) | 18.91  (17.51-20.39) | 20.16  (18.71-21.69) | 18.32  (16.86-19.86) | 19.91  (18.45-21.46) |
|  | Q3 | 33.10  (31.96-34.28) | 34.05  (32.88-35.24) | 32.45  (31.31-33.62) | 31.64  (30.51-32.79) | 48.00  (46.09-49.95) | 50.28  (48.34-52.28) | 47.14  (45.25-49.08) | 44.15  (42.32-46.03) | 17.03  (15.84-18.29) | 16.67  (15.47-17.94) | 17.05  (15.83-18.33) | 18.58  (17.30-19.93) |
|  | Q4 | 27.30  (26.36-28.27) | 28.28  (27.31-29.27) | 26.03  (25.10-26.98) | 23.95  (23.05-24.88) | 39.81  (38.26-41.41) | 39.79  (38.24-41.38) | 36.23  (34.77-37.74) | 32.23  (30.83-33.67) | 13.77  (12.78-14.81) | 15.49  (14.41-16.61) | 14.69  (13.63-15.81) | 14.72  (13.63-15.86) |
|  | Q5 | 21.41  (20.59-22.26) | 22.75  (21.87-23.65) | 21.87  (21.01-22.76) | 20.87  (20.01-21.75) | 30.23  (28.88-31.62) | 31.18  (29.80-32.61) | 29.21  (27.87-30.60) | 27.97  (26.63-29.36) | 12.19  (11.27-13.15) | 14.09  (13.05-15.18) | 14.24  (13.20-15.34) | 13.16  (12.16-14.22) |
| Inequality | |  |  |  |  |  |  |  |  |  |  |  |  |
| SII | | 33.40  (31.40-35.44) | 32.16  (30.14-34.22) | 35.34  (33.30-37.45) | 35.32  (33.29-37.39) | 54.23  (50.86-57.68) | 55.12  (51.72-58.64) | 57.12  (53.75-60.59) | 56.65  (53.29-60.12) | 17.40  (15.16-19.76) | 14.25  (12.00-16.56) | 17.93  (15.53-20.40) | 18.75  (16.42-21.21) |
| RI | | 3.20  (2.95-3.48) | 2.91  (2.70-3.15) | 3.48  (3.19-3.81) | 3.68  (3.35-4.05) | 3.74  (3.39-4.17) | 3.69  (3.35-4.10) | 4.35  (3.89-4.91) | 4.76  (4.21-5.44) | 3.10  (2.63-3.72) | 2.32  (2.01-2.71) | 2.90  (2.47-3.45) | 3.08  (2.62-3.67) |
| RD (Q1-Q5) | | 16.16 | 15.63 | 20.78 | 19.11 | 31.09 | 31.18 | 37.38 | 34.55 | 6.64 | 5.21 | 8.18 | 8.40 |
| RR (Q1/Q5) | | 1.75 | 1.69 | 1.95 | 1.92 | 2.03 | 2.00 | 2.28 | 2.24 | 1.54 | 1.37 | 1.57 | 1.64 |

Values of Q0-Q5 are presented as ASMR per 100,000 population (95% confidence interval).
ASMR, age-standardized mortality rate; SII, slope index of inequality; RII, relative index of inequality; RD, rate difference; RR, rate ratio; Q0, Medicaid beneficiaries; Q1-Q5, quintile of national health insurance premiums
