# Supplementary material for: Did the socioeconomic inequalities in avoidable and unavoidable mortality worsen during the first year of the COVID-19 pandemic in Korea?
Source: Epidemiol Health. 2023 Aug 3;45:e2023072. doi: 10.4178/epih.e2023072 (PMC10728611; doi:10.4178/epih.e2023072)
Supplement: Supplement Material 7. — Annual ASMRs and absolute and relative inequality of ASMRs due to diseases of the circulatory system from 2017 to 2020 [file epih-45-e2023072-Supplementary-7.docx]

Supplementary Material 7. Annual ASMRs and absolute and relative inequality of ASMRs due to diseases of the circulatory system from 2017 to 2020

|  | | All | | | | Men | | | | Women | | | |
| --- | --- | --- | --- | --- | --- | --- | --- | --- | --- | --- | --- | --- | --- |
|  |  | 2017 | 2018 | 2019 | 2020 | 2017 | 2018 | 2019 | 2020 | 2017 | 2018 | 2019 | 2020 |
| ASMR (/100,000) | |  |  |  |  |  |  |  |  |  |  |  |  |
|  | Q0 | 90.75  (86.06-95.60) | 90.03  (85.39-94.82) | 89.46  (84.62-94.44) | 84.52  (80.03-89.14) | 137.25  (128.78-146.04) | 133.49  (125.62-141.63) | 132.11  (123.39-141.15) | 123.24  (115.55-131.19) | 45.84  (41.25-50.71) | 47.14  (41.90-52.70) | 46.17  (41.34-51.30) | 43.69  (38.90-48.79) |
|  | Q1 | 19.68  (18.81-20.57) | 18.60  (17.79-19.44) | 18.44  (17.67-19.23) | 17.87  (17.10-18.66) | 31.82  (30.17-33.53) | 29.89  (28.34-31.50) | 29.54  (28.11-31.03) | 28.18  (26.75-29.67) | 10.10  (9.26-10.99) | 9.52  (8.74-10.35) | 9.10  (8.35-9.89) | 9.49  (8.73-10.29) |
|  | Q2 | 18.97  (18.08-19.90) | 17.98  (17.12-18.87) | 16.70  (15.83-17.60) | 17.61  (16.76-18.49) | 27.93  (26.40-29.51) | 26.74  (25.25-28.28) | 25.61  (24.06-27.23) | 27.24  (25.72-28.82) | 10.03  (9.09-11.05) | 9.48  (8.57-10.45) | 8.41  (7.52-9.36) | 8.66  (7.81-9.57) |
|  | Q3 | 18.57  (17.75-19.42) | 16.97  (16.19-17.77) | 16.17  (15.43-16.94) | 15.64  (14.92-16.37) | 26.71  (25.33-28.15) | 23.84  (22.55-25.18) | 23.50  (22.24-24.80) | 23.17  (21.95-24.44) | 10.26  (9.39-11.18) | 9.92  (9.08-10.82) | 8.69  (7.92-9.51) | 8.04  (7.33-8.81) |
|  | Q4 | 16.11  (15.45-16.78) | 15.12  (14.50-15.76) | 13.34  (12.76-13.94) | 13.07  (12.49-13.66) | 22.61  (21.53-23.74) | 21.63  (20.59-22.71) | 19.17  (18.20-20.17) | 18.34  (17.40-19.32) | 9.59  (8.87-10.35) | 8.51  (7.86-9.21) | 7.32  (6.71-7.97) | 7.46  (6.85-8.11) |
|  | Q5 | 11.83  (11.33-12.34) | 11.25  (10.76-11.75) | 10.54  (10.09-11.02) | 9.68  (9.24-10.14) | 16.99  (16.13-17.88) | 16.53  (15.68-17.41) | 14.94  (14.17-15.75) | 14.00  (13.25-14.79) | 7.13  (6.61-7.68) | 6.45  (5.95-6.98) | 6.48  (5.98-7.01) | 5.57  (5.11-6.07) |
| Inequality | |  |  |  |  |  |  |  |  |  |  |  |  |
| SII | | 21.06  (19.81-22.34) | 20.66  (19.44-21.90) | 20.75  (19.56-21.99) | 21.04  (19.88-22.22) | 33.91  (31.72-36.18) | 32.20  (30.06-34.38) | 33.63  (31.50-35.80) | 33.54  (31.53-35.62) | 9.29  (8.00-10.63) | 10.00  (8.69-11.38) | 8.94  (7.72-10.23) | 9.92  (8.71-11.17) |
| RII | | 3.74  (3.39-4.13) | 3.97  (3.59-4.43) | 4.51  (4.03-5.09) | 4.97  (4.41-5.65) | 4.52  (3.97-5.19) | 4.50  (3.95-5.19) | 5.69  (4.88-6.75) | 6.15  (5.25-7.37) | 2.69  (2.31-3.16) | 3.20  (2.69-3.87) | 3.08  (2.58-3.73) | 3.79  (3.12-4.71) |
| RD (Q1-Q5) | | 7.85 | 7.35 | 7.90 | 8.19 | 14.83 | 13.36 | 14.60 | 14.18 | 2.97 | 3.07 | 2.62 | 3.92 |
| RR (Q1/Q5) | | 1.66 | 1.65 | 1.75 | 1.85 | 1.87 | 1.81 | 1.98 | 2.01 | 1.42 | 1.48 | 1.40 | 1.70 |

Values of Q0-Q5 are presented as ASMR per 100,000 population (95% confidence interval).
ASMR, age-standardized mortality rate; SII, slope index of inequality; RII, relative index of inequality; RD, rate difference; RR, rate ratio; Q0, Medicaid beneficiaries; Q1-Q5, quintile of national health insurance premiums
