# Supplementary material for: Did the socioeconomic inequalities in avoidable and unavoidable mortality worsen during the first year of the COVID-19 pandemic in Korea?
Source: Epidemiol Health. 2023 Aug 3;45:e2023072. doi: 10.4178/epih.e2023072 (PMC10728611; doi:10.4178/epih.e2023072)
Supplement: Supplement Material 8. — Annual ASMRs and absolute and relative inequality for suicides from 2017 to 2020 [file epih-45-e2023072-Supplementary-8.docx]

Supplementary Material 8. Annual ASMRs and absolute and relative inequality for suicides from 2017 to 2020

|  | | All | | | | Men | | | | Women | | | |
| --- | --- | --- | --- | --- | --- | --- | --- | --- | --- | --- | --- | --- | --- |
|  |  | 2017 | 2018 | 2019 | 2020 | 2017 | 2018 | 2019 | 2020 | 2017 | 2018 | 2019 | 2020 |
| ASMR (/100,000) | |  |  |  |  |  |  |  |  |  |  |  |  |
|  | Q0 | 58.64  (52.78-64.83) | 54.40  (49.00-60.10) | 59.67  (53.61-66.05) | 57.35  (51.46-63.57) | 80.62  (70.83-91.04) | 74.09  (65.02-83.77) | 72.24  (62.96-82.13) | 69.73  (60.94-79.09) | 38.71  (31.76-46.40) | 36.30  (30.02-43.24) | 47.05  (39.19-55.68) | 45.69  (37.68-54.52) |
|  | Q1 | 23.11  (22.01-24.24) | 24.45  (23.35-25.59) | 28.60  (27.48-29.76) | 26.45  (25.33-27.59) | 35.94  (33.93-38.04) | 39.08  (37.03-41.22) | 43.46  (41.47-45.52) | 39.35  (37.38-41.39) | 13.06  (11.93-14.28) | 12.92  (11.80-14.12) | 16.21  (15.01-17.48) | 16.11  (14.87-17.41) |
|  | Q2 | 22.32  (21.28-23.40) | 25.00  (23.87-26.15) | 21.63  (20.53-22.77) | 23.95  (22.83-25.12) | 33.32  (31.50-35.22) | 37.49  (35.52-39.53) | 31.34  (29.42-33.35) | 34.38  (32.41-36.42) | 12.29  (11.19-13.48) | 13.88  (12.70-15.15) | 13.73  (12.49-15.06) | 15.18  (13.92-16.52) |
|  | Q3 | 18.52  (17.67-19.40) | 20.87  (19.96-21.82) | 20.33  (19.43-21.26) | 20.91  (19.99-21.87) | 25.83  (24.45-27.27) | 29.76  (28.27-31.31) | 28.28  (26.83-29.79) | 27.67  (26.21-29.18) | 10.59  (9.65-11.59) | 11.33  (10.33-12.39) | 11.98  (10.95-13.07) | 13.89  (12.77-15.08) |
|  | Q4 | 15.23  (14.51-15.97) | 17.40  (16.63-18.20) | 16.74  (15.99-17.53) | 15.07  (14.34-15.84) | 20.85  (19.72-22.03) | 23.18  (21.98-24.42) | 21.78  (20.63-22.97) | 18.59  (17.51-19.72) | 9.05  (8.23-9.93) | 10.84  (9.93-11.81) | 10.98  (10.05-11.97) | 11.05  (10.08-12.08) |
|  | Q5 | 12.90  (12.24-13.59) | 14.35  (13.63-15.10) | 14.20  (13.49-14.94) | 14.03  (13.30-14.78) | 17.30  (16.26-18.39) | 18.55  (17.45-19.70) | 17.71  (16.63-18.82) | 17.57  (16.47-18.72) | 8.26  (7.48-9.09) | 10.00  (9.09-10.96) | 10.50  (9.57-11.48) | 10.06  (9.15-11.02) |
| Inequality | |  |  |  |  |  |  |  |  |  |  |  |  |
| SII | | 19.32  (17.74-20.94) | 18.99  (17.36-20.64) | 22.00  (20.32-23.69) | 21.64  (20.00-23.35) | 30.97  (28.37-33.69) | 33.12  (30.45-35.89) | 35.04  (32.37-37.79) | 33.61  (30.99-36.33) | 10.49  (8.68-12.43) | 8.14  (6.26-10.11) | 11.79  (9.76-13.95) | 12.76  (10.69-14.94) |
| RII | | 3.18  (2.85-3.57) | 2.75  (2.49-3.04) | 3.32  (2.98-3.72) | 3.35  (3.00-3.77) | 3.93  (3.40-4.59) | 3.75  (3.29-4.31) | 4.42  (3.82-5.20) | 4.47  (3.84-5.29) | 2.81  (2.30-3.52) | 2.00  (1.69-2.40) | 2.60  (2.17-3.18) | 2.74  (2.29-3.36) |
| RD (Q1-Q5) | | 10.21 | 10.10 | 14.40 | 12.42 | 18.64 | 20.53 | 25.75 | 21.78 | 4.8 | 2.92 | 5.71 | 6.05 |
| RR (Q1/Q5) | | 1.79 | 1.70 | 2.01 | 1.89 | 2.08 | 2.11 | 2.45 | 2.24 | 1.58 | 1.29 | 1.54 | 1.60 |

Values of Q0-Q5 are presented as ASMR per 100,000 population (95% confidence interval).
ASMR, age-standardized mortality rate; SII, slope index of inequality; RII, relative index of inequality; RD, rate difference; RR, rate ratio; Q0, Medicaid beneficiaries; Q1-Q5, quintile of national health insurance premiums
